# Effects of Music Therapy on Patients with Dementia—A Systematic Review

**DOI:** 10.3390/geriatrics5040062

**Published:** 2020-09-25

**Authors:** Hei Long Lam, Wai Tak Victor Li, Ismail Laher, Roger Y. Wong

**Affiliations:** 1 Li Ka Shing Faculty of Medicine, University of Hong Kong, Hong Kong SAR, China; aaronlhl@hku.hk (H.L.L.); u3548592@hku.hk (W.T.V.L.); 2 Department of Anesthesiology, Pharmacology and Therapeutics, Faculty of Medicine, University of British Columbia, Vancouver, BC V6T 1Z3, Canada; ismail.laher@ubc.ca; 3 Department of Medicine, Faculty of Medicine, University of British Columbia, Vancouver, BC V5Z 1M9, Canada

**Keywords:** music therapy, dementia, systematic review

## Abstract

Dementia is an increasingly common syndrome and while pharmacotherapy is available, its potential benefit is limited, especially in non-cognitive outcomes. Non-pharmacotherapy such as music therapy is potentially associated with improved outcomes. We assessed the effects of music therapy on patients with dementia to evaluate its potential benefits on dementia. Two independent reviewers searched MEDLINE, EMBASE, CINAHL, CENTRAL, and ClinicalTrials.gov databases for clinical trials, using the keywords “music therapy” and “dementia”. Study outcomes included cognitive function, behavioral and psychological symptoms of dementia (BPSD), and quality of life. A total of 82 studies were included, of which 43 were interventional clinical trials, and 39 were systematic reviews or meta-analyses. Significant improvements in verbal fluency occurred after music therapy, with significant reductions in anxiety, depression, and apathy. There were no significant improvements in cognition or daily functioning, and the results on quality of life and agitation were ambiguous. Limitations of studies included low patient numbers, lack of standardized music therapy, and high heterogeneity in outcomes. More large-scale clinical trials would allow for clearer conclusions on the benefits of music therapy in patients with dementia.

## 1. Introduction

The aging population is increasing rapidly, and it is predicted that one out of six people will be aged 65 years and over in 2050 [1]. Dementia is a not part of normal aging, but it has gained increased awareness globally, as summarized in a recent systematic review on the prevalence and incidence of dementia [2]. In addition to age, other risk factors for dementia include family history (genetics), cardiovascular risks (e.g., hypertension, dyslipidemia, cigarette smoking, stroke), and female gender [3].

According to the American Psychiatric Association’s Diagnostic and Statistical Manual, Fifth Edition (DSM-5), dementia is a major neurocognitive disorder that is diagnosed when one or more cognitive domains, such as complex attention, executive ability, learning and memory, language, praxis, and social cognition, are impaired. It should be noted that dementia is not a disease, but rather a syndrome that can present in different forms such as Alzheimer’s disease, vascular dementia, mixed Alzheimer’s disease and cerebrovascular disease, and Lewy body dementia. Alzheimer’s disease accounts for 60% of dementia cases [4]. The standard pharmacological treatment for dementia is cholinesterase inhibitors, which can delay the deterioration of cognitive function. However, cholinesterase inhibitors have limited efficacy with small improvements of symptoms related to non-cognitive outcomes [5]. Music therapy is used as an alternative or adjunct treatment in some cases of dementia [6].

Music therapy has a long history, where the first documented music therapy session consisted of a group of musicians performing in patients’ wards; benefits included reduced pain and “calming and stimulating effects”, as quoted by hospital staff members [7]. According to the American Music Therapy Association, music therapy is defined as “the clinical and evidence-based use of music interventions to accomplish individualized goals within a therapeutic relationship by a credentialed professional who has completed an approved music therapy program” [8].

Music therapy can be delivered in several modalities, as shown in Table 1, and can be divided into active participation (playing instruments/singing) or passive participation (listening). The therapy can be carried out at home or in aged care residences with either one-on-one or group sessions.

This review assesses the effects of music therapy on patients with dementia by considering outcomes explored in clinical trials.

## 2. Materials and Methods

We searched MEDLINE, EMBASE, CINAHL, CENTRAL, and ClinicalTrials.gov databases for clinical trials reported from 1 January 1946 up to 1 May 2020. Search keywords included “music therapy” and “dementia”. A detailed search strategy for MEDLINE and EMBASE via Ovid is presented in Appendix A. Search filters suggested by the British Medical Journal’s Knowledge Centre and CareSearch were used to maximize sensitivity. To further increase the sensitivity of our search, the reference lists from previously published reviews on music therapy and dementia were also searched.

All clinical trials were used to create a more comprehensive review. These included interventional trials (such as randomized controlled trials) and observational trials (such as cohort studies, case-control studies, case series, and case studies). Systematic reviews and meta analyses of clinical trials were also included. All study outcomes were included, provided that the study population was clinically diagnosed with any stage of dementia. Primary study outcomes included cognitive, behavioral, and psychological symptoms of dementia (BPSD), including anxiety, depression, agitation, and apathy. Secondary outcomes included daily functioning, physiological outcomes, and quality of life. While music therapy might be implemented in various forms such as individualized or group sessions with active or passive participation, all types of music therapy were included in this review.

Studies with music therapy combined with other interventions (meditation, drawing, etc.) as the primary intervention, were excluded, for increased accuracy. Studies with patients having unclear clinical diagnosis of dementia were also excluded. Studies with rhythmic auditory stimulation as intervention were excluded. The inclusion and exclusion criteria are shown in Table 2. Screening of eligible publications was carried out by two independent reviewers (HLL and WTVL), with the search results exported to and de-duplicated by Endnote X9. Disagreements were reviewed by a third independent reviewer (IL) and were resolved through discussion.

**Figure 1:**
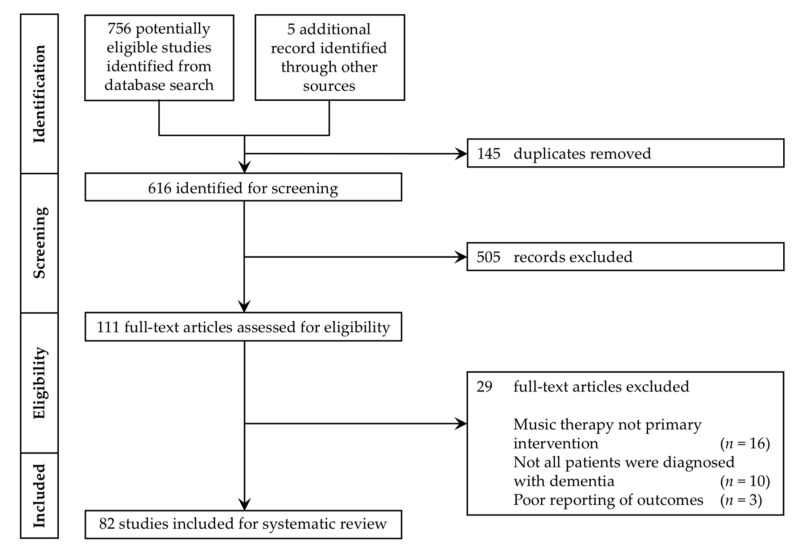
Preferred Reporting Items for Systematic Reviews and Meta-Analyses (PRISMA) flowchart.

After the removal of duplicate studies, 616 records were screened, and 82 studies were included in this review. A flowchart of the selection process is shown in Figure 1.

Among the 82 included studies, 43 were interventional clinical trials and 39 were systematic reviews or meta-analyses. Hence, the results were synthesized from the 43 trials. For each study, the population, modality of music intervention, control group, and outcomes were extracted in a standardized manner, using Microsoft Excel. The data were coded by categorizing each outcome in the included studies into “significant”, “mixed”, or “insignificant” effects. The findings in turn populated the results table (Table 3), according to the modality of the music therapy and outcomes.

## 3. Results

### 3.1. Cognition

#### 3.1.1. Overall Cognition

Thirteen studies reported overall cognition as an outcome, with 4 showing significant improvements and 8 reporting no significant improvements. One study reported a mixed effect, as only improvement in the abstraction domain of cognition was observed [10].

In one RCT involving 30 elderly patients that explored the effects of song-writing [11], patients in the experimental group showed significant increases in the MMSE (Korean adaptation) score, with significant improvements in language function, orientation, and memory scores.

However, another RCT showed no significant improvement in cognition when 59 patients were randomly assigned to a music therapy group or a control group [12]. The music therapy group used musical instruments in a nonverbal environment with a music therapist. The findings suggested that playing musical instruments does not improve cognition in patients living with dementia. Another study that explored the effects of lyric reading produced similar findings [13].

Results were mixed for the effects of other forms of music therapy on the cognitive status in patients living with dementia. In one RCT, listening to music only improved the abstraction domain after adjustments for sex, age, education level, neuropsychiatric inventory, and corresponding baseline measurements [10]. For music therapy involving two or more modalities, such as both listening to music and singing, only one out of four studies reported improvements in the overall cognition in the intervention group [14].

#### 3.1.2. Memory

Results for the effect of music therapy on the memory of patients living with dementia were mixed, with 4 out of 5 studies reporting significant improvements. Two RCTs assessed the effects of singing on memory; one study reported significant improvements in memory, as measured by a comprehensive battery of neuropsychological tests [15], while the other study found no significant differences between the intervention and control groups when the Mini-Mental State Examination was used as an assessment tool [13]. It is worth noting that significant improvements in memory occurred in the two studies that used a combined music therapy modality [16,17].

#### 3.1.3. Language

All 3 RCTs with language or verbal fluency as an outcome reported significant improvements in the music therapy groups [11,13,18]. Music therapy modalities that improved language performance included singing, song writing, lyric reading, and combined music therapy.

### 3.2. Behavioral and Psychological Symptoms of Dementia (BPSDs)

#### 3.2.1. Overall BPSD

Both RCTs that explored the effects of singing on BPSD showed significant reductions in BPSD in patients living with dementia, as measured by the neuropsychiatric inventory [13,19]. Two RCTs that used a combined music therapy modality also reported reduced overall BPSD [20,21]. However, results were mixed for music listening and using musical instruments.

#### 3.2.2. Anxiety and Depression

Most of the included studies showed a significant reduction in either anxiety or depression, or both. Among the 7 studies with music listening as the primary intervention, 5 studies reported that music listening significantly improved mood, while no significant effect was observed in the remaining 2 studies. All but one study from the 7 studies that used a combined music therapy modality showed significantly improved mood in the music therapy group. An RCT involving 59 patients reported a significant reduction in anxiety measured by the State Trait Anxiety Inventory, but no significant reduction in depression when measured by the Geriatric Depression Scale [22].

#### 3.2.3. Agitation

There were mixed results for the studies of the effect of music therapy on agitation, in patients living with dementia. While 6 out of 8 studies reported significantly reduced agitation when music listening was the primary intervention, mixed results were observed for the studies exploring the effects of a combined music therapy, in which music listening was also frequently involved. It is worth noting that in a crossover trial in which Baroque music was played to 75 patients, an adverse effect was observed where there was a significantly increased number of episodes of agitated behavior when music was played [23].

### 3.3. Apathy

Levels of apathy were reduced in the two of the trials in which active music therapy was implemented (using musical instruments and combined music therapy respectively) in 137 patients [24,25]. Another study exploring the effects of music listening reported that apathy was significantly reduced in the intervention group in which live music was played, but no significant effect was observed for the group in which pre-recorded music was played (need reference for this study). This suggests that patient participation might be a factor affecting apathy.

### 3.4. Daily Functioning

Two studies with daily functioning as an outcome reported no significant improvements in the music therapy group [26,27]. Music therapy modalities in these trials included singing, playing musical instruments, and lyric reading.

### 3.5. Physiological Outcomes

A non-randomized controlled trial reported that music therapy significantly lowered plasma interleukin-6 and catecholamine levels, in addition to significantly lowering the complications of congestive heart failure [28]. Mixed results were reported on the relationship between music therapy and salivary cortisol levels, which is a marker of stress.

### 3.6. Quality of Life

Mixed results were reported on the effects of music therapy on the quality of life. Four trials reported significant improvements while two trials did not observe significant effects.

## 4. Discussion

Our systematic review of studies on the effects of music therapy of patients living with dementia suggests significant improvements in verbal and language fluency, alleviation of BPSD including anxiety and depression and reduced levels of apathy. These findings are generally in line with previous reports on the potential benefits of music therapy in improving the behavioral symptoms in patients living with dementia [53,54]. However, our study suggests that music therapy failed to significantly improve the overall aspects of cognition (such as memory, orientation, and registration), agitation, daily functioning, and the quality of life of patients living with dementia.

The observed benefits of music therapy in improving verbal and language fluency could have a biological basis. Verbal production is mediated by a language output system that is neurologically distinct from the melodic output system [55]. Nonetheless, recall of familiar melodies with lyrics is preserved in patients who cannot recall melodies without lyrics, after experiencing a stroke [56]. This suggests the simultaneous formation of integrated memories of melodies and lyrics. Coincidently, the modalities of music therapy used in studies that show improvement in verbal and language fluency all included a language component. This might suggest that lyrics in pieces used for music therapy could play an important role in memory formation activation, and hence improve verbal fluency in patients living with dementia.

The ability of music therapy to alleviate anxiety and depression is apparent in the selected groups of patients [57,58]. This finding is in keeping with a previous meta-analysis on patients living with dementia [59]. Apathy was also reduced, which was in agreement with a previous meta-analysis where patients living with dementia demonstrated improved apathy after music therapy [60]. However, many studies did not specify the type of music used; the selection of music might therefore be subject to personal bias—for example, the authors might choose happier or more light-hearted music and influence symptom alleviation.

Interestingly, our review shows that overall cognition and related aspects such as memory, orientation, and registration, are not significantly improved by music therapy. One possible explanation is that the improved cognition reported in the literature could be mostly due to an improvement in verbal and language fluency, although this would require confirmation by future studies. While most forms of music might be helpful in relieving BPSD, there might be subtle differences with some types of music, such as Baroque music, which might be more musically activating and complex, and which could inadvertently increase the agitation levels in patients [13]. The assessment of daily functioning and quality of life is largely dependent on the motor skills and intention of action in patients living with dementia, neither of which is the focus of music therapy. The short-term benefits of music therapy in alleviating BPSD, possibly due to inducing comfort and emotional safety [61], might not sustain in the long-term and, hence might not improve the quality of life of patients living with dementia.

The extrapolation of our review findings could be difficult due to the highly heterogeneous study designs used in the different trials. Music therapies used in the studies demonstrate large variations in duration, genres of music, and forms of appreciation. This makes it difficult for us to consistently assess the quality of individual studies. Practically, however, such heterogeneity makes sense, as music encompasses a broad spectrum and music therapy of any nature should be tailored to generalized patient goals, as recommended by the American Music Therapy Association [62].

Recent systematic reviews and meta-analyses that investigated the effects of music therapy suggested that music therapy could be useful in improving the domains of memory [63] and agitation [64], in patients living with dementia. Our review findings differed in both domains, as our criterion was more stringent because we reported the overall effect of music therapy as mixed when there was at least one study showing statistically insignificant trends. Interestingly, one of these recent reviews found supportive evidence for the use of music therapy in alleviating BPSD and anxiety in patients living with dementia, similar to our findings [53].

There were a number of additional limitations in our study. First, most studies did not distinguish between depression, agitation, or apathy as conditions secondary to dementia, or as independently coexisting conditions with dementia. This might produce a confounding effect. Second, it was unclear if alleviations of symptoms were attributed to the therapeutic effects of music, or if the changes were a part of the natural disease progression of dementia. For example, symptoms of depression might be alleviated, as the severity of dementia worsened in some patients. Third, music therapy sessions that were generalized or administered in groups were not differentiated in the analysis and extrapolation of results. This might overlook the benefits of each of these modes of administration, for example the increased social interaction in group interventions versus the tailor-made choice of music to meet individual needs. Fourth, the outcomes reported were measured using different tools or scales (see Results). These assessments were often not performance-based or made by clinicians, but rather were observation-based by carers of these patients, thus, introducing possible reporting bias. That said, caregiver stress or burden was not specifically measured in the studies. Fifth, due to the highly heterogeneous study designs and reporting of results, quality assessment of each included study was not performed, as there was no single tool that could comprehensively evaluate all of these studies. Lastly, the results on physiological outcomes were difficult to generalize as the clinical effects of such surrogate markers were difficult to interpret in different settings.

The results of our review have two important implications in informing best practices. First, the alleviation of BPSD in patients living with dementia who received music therapy might suggest the reduction of caregiver stress or burden, as suggested by the included studies, as well as other evidence in the literature [13,15,20,31,65]. However, this observation was not an outcome set in our review, hence, it warranted confirmation from further independent studies. Second, the benefits of music therapy could be potentially complimentary to pharmacotherapy in patients living with dementia. Pharmacotherapy, such as with cholinesterase inhibitors, was associated with improvement in cognitive function and daily activities [66,67], whereas music therapy could be associated with improvement in verbal fluency and reductions in anxiety, depression, and apathy. Further studies are warranted to investigate the combined effects of pharmacotherapy and music therapy in the care of older adults living with dementia.

## 5. Conclusions

Music therapy could improve verbal fluency and reduce anxiety, depression, and apathy in selected patients living with dementia, although there does not appear to be proven benefits on memory, daily function, or overall quality of life. More clinical trials are needed to allow for more definitive conclusions on the therapeutic value of music therapy to patients with dementia.

## Figures and Tables

**Table 1:** Modalities of music therapy.

Modalities [9]	Description
Song writing	Usually under the guidance of a music therapist in a group session; patients write lyrics for a melody.
Directed music listening	Music is played to patients in individualized sessions, sometimes according to their preference.
Music and relaxation exercises	Patients stretch and breathe to the rhythm of music.
Lyric discussion	A group-based session where patients discuss the lyrics of a song with each other, often in the presence of a music therapist.
Singing/Toning	Patients sing to accompany a given melody with lyrics.
Moving to music	Patients move or dance to songs.
Recording and video creation	Patients participate in producing a music recording or video.
Adapted instrument lessons	Patients learn to play musical instruments with the difficulty adapted to their personal condition.

**Table 2:** Inclusion and exclusion criteria of studies.

Inclusion Criteria of Study:	Exclusion Criteria of Study:
Types of study: Randomized controlled trials (RCTs), cohort studies, case-control studies, case reports and case series, systematic reviews and meta analyses	Studies with music therapy combined with another intervention (e.g., cognitive enhancement therapies)
English full-text articles only	Studies with patients with unclear/possible clinical diagnosis of dementia, or patients with mild cognitive impairment
Study intervention: music therapy	Interventions of rhythmic auditory stimulation
Study population: patients clinically diagnosed with dementia	

**Table 3:** Summary of studies on music therapy on outcomes.

	Modality of Music Therapy	Singing	Music Listening	Playing Musical Instruments	Song-Writing	Lyric Reading	Combined (2 or More Modalities)
Outcomes							
Cognition							
Improved overall cognition		Y: Two studies [15,19] N: One study [13]	Y: One study [15] Mixed: One study [10] * N: Two studies [29,30]	N: One study [12]	Y: One study [11]	N: One study [13]	Y: One study [14] N: Three studies [18,25,31]
Improved memory		Y: One study [15] N: One study [13]	–	–	Y: One study [11]	N: One study [13]	Y: Two studies [16,17]
Improved language or verbal fluency		Y: One study [13]	–	–	Y: One study [11]	Y: One study [13]	Y: One study [18]
Behavioral and Psychological Symptoms of Dementia (BPSD)							
Reduced overall BPSD (lower NPI score)		Y: Two studies [13,25]	Y: Two studies [32,33] N: Three studies [10,34,35]	Y: One study [12] N: One study [35]	–	Y: One study [13]	Y: Two studies [20,21]
Reduced anxiety or depression		Mixed: One study [22] **	Y: Five studies [29,36,37,38,39] N: Two studies [30,40]	N: One study [24]	–	–	Y: Six studies [14,17,20,27,31,41] N: One study [42,43] ****
Reduced agitation		–	Y: Six studies [29,30,40,44,45,46] N: Two studies [23,34]	Y: One study [24]	–	–	Y: Five studies [18,26,31,47,48] N: Four studies [41,42,49,50]
Apathy							
Reduced apathy		–	Mixed: One study [51] ***	Y: One study [24]	–	–	Y: One study [25]
Daily Functioning							
Improved daily functioning (improved Barthel Index)		N: One study [13]	–	N: One study [12]	–	N: One study [13]	Y: One study [16]
Physiological outcomes							
Reduced congested heart failure events		–	–	–	–	–	Y: One study [28]
Reduced pain		Y: One study [22]	–	–	–	–	–
Increased appetite		–	Y: One study [38]	–	–	–	–
Lowered blood pressure		–	–	–	–	–	Y: One study [52]
Decreased IL-6 and catecholamines		–	–	–	–	–	Y: One study [28]
Reduction in salivary cortisol		–	–	–	–	–	Y: One study [27] N: Two studies [14,52]
Quality of life							
Improved quality of life		Y: One study [22]	Y: One study [15] N: One study [33]	–	–	–	Y: Two studies [20,33] N: Two studies [43,48]

Y: yes for significant improvement; mixed: mixed effect in the improvement of different sub-categories of measurement; N: no significant improvement. * Music therapy group only showed a significant improvement in abstraction domain after adjustment. ** Anxiety was significantly reduced while no significant effect was observed for depression. *** Live music reduced apathy while pre-recorded music did not. **** [42,43] are from one single trial.

## Appendix A

*Search strategy for Medline* via *Ovid*

1. review.pt.

2. (medline or medlars or embase or pubmed or cochrane).tw,sh.

3. (scisearch or psychinfo or psycinfo).tw,sh.

4. (psychlit or psyclit).tw,sh.

5. cinahl.tw,sh.

6. ((hand adj2 search$) or (manual$ adj2 search$)).tw,sh.

7. (electronic database$ or bibliographic database$ or computeri?ed database$ or online database$).tw,sh.

8. (pooling or pooled or mantel haenszel).tw,sh.

9. (peto or dersimonian or der simonian or fixed effect).tw,sh.

10. (retraction of publication or retracted publication).pt.

11. or/2–10

12. 1 and 11

13. meta-analysis.pt.

14. meta-analysis.sh.

15. (meta-analys$ or meta analys$ or metaanalys$).tw,sh.

16. (systematic$ adj5 review$).tw,sh.

17. (systematic$ adj5 overview$).tw,sh.

18. (quantitativ$ adj5 review$).tw,sh.

19. (quantitativ$ adj5 overview$).tw,sh.

20. (quantitativ$ adj5 synthesis$).tw,sh.

21. (methodologic$ adj5 review$).tw,sh.

22. (methodologic$ adj5 overview$).tw,sh.

23. (integrative research review$ or research integration).tw.

24. or/13–23

25. 12 or 24

26. randomized controlled trial.pt.

27. (random$ or placebo$ or single blind$ or double blind$ or triple blind$).ti,ab.

28. (retraction of publication or retracted publication).pt.

29. or/26–28

30. (animals not humans).sh.

31. ((comment or editorial or meta-analysis or practice-guideline or review or letter) not randomized controlled trial).pt.

32. (random sampl$ or random digit$ or random effect$ or random survey or random regression).ti,ab. not randomized controlled trial.pt.

33. 29 not (30 or 31 or 32)

34. exp cohort studies/

35. cohort$.tw.

36. controlled clinical trial.pt.

37. epidemiologic methods/

38. limit 4 to yr=1966–1989

39. exp case-control studies/

40. (case$ and control$).tw.

41. (case$ and series).tw.

42. case reports.pt.

43. (case$ adj2 report$).tw.

44. (case$ adj2 stud$).tw.

45. or/34–36,38–44

46. exp music therapy/

47. exp music/

48. exp dementia/ or dement$.ti,ab. or alzheimer$.mp.

49. 46 or 47

50. 25 or 33 or 45

51. 48 and 49 and 50

*Search strategy for Embase* via *Ovid*

1. exp cohort analysis/

2. exp longitudinal study/

3. exp prospective study/

4. exp follow up/

5. cohort$.tw.

6. exp case control study/

7. (case$ and control$).tw.

8. exp case study/

9. (case$ and series).tw.

10. case report/

11. (case$ adj2 report$).tw.

12. (case$ adj2 stud$).tw.

13. or/1–12

14. (random$ or placebo$ or single blind$ or double blind$ or triple blind$).ti,ab.

15. RETRACTED ARTICLE/

16. or/14–15

17. (animal$ not human$).sh,hw.

18. (book or conference paper or editorial or letter or review).pt. not exp randomized controlled trial/

19. (random sampl$ or random digit$ or random effect$ or random survey or random regression).ti,ab. not exp randomized controlled trial/

20. 16 not (17 or 18 or 19)

21. exp review/

22. (literature adj3 review$).ti,ab.

23. exp meta analysis/

24. exp systematic review/

25. or/21–24

26. (medline or medlars or embase or pubmed or cinahl or amed or psychlit or psyclit or psychinfo or psycinfo or scisearch or cochrane).ti,ab.

27. 15 or 26

28. 25 and 27

29. (systematic$ adj2 (review$ or overview)).ti,ab.

30. (meta?anal$ or meta anal$ or meta-anal$ or metaanal$ or metanal$).ti,ab.

31. 28 or 29 or 30

32. exp dementia/ or dement$.ti,ab. or alzheimer$.mp.

33. exp music therapy/

34. exp music/

35. 33 or 34

36. 13 or 20 or 31

37. 32 and 35 and 3
